# Proteome-wide measurement of non-canonical bacterial mistranslation by quantitative mass spectrometry of protein modifications

**DOI:** 10.1038/srep28631

**Published:** 2016-07-05

**Authors:** Nevena Cvetesic, Maja Semanjski, Boumediene Soufi, Karsten Krug, Ita Gruic-Sovulj, Boris Macek

**Affiliations:** 1 Chemistry Department, Faculty of Science, University of Zagreb, Croatia; 2 Proteome Center Tuebingen, University of Tuebingen, Germany

## Abstract

The genetic code is virtually universal in biology and was likely established before the advent of cellular life. The extent to which mistranslation occurs is poorly understood and presents a fundamental question in basic research and production of recombinant proteins. Here we used shotgun proteomics combined with unbiased protein modification analysis to quantitatively analyze *in vivo* mistranslation in an *E. coli* strain with a defect in the editing mechanism of leucyl-tRNA synthetase. We detected the misincorporation of a non-proteinogenic amino acid norvaline on 10% of all measured leucine residues under microaerobic conditions and revealed preferential deployment of a tRNA^Leu^(CAG) isoacceptor during norvaline misincorporation. The strain with the norvalylated proteome demonstrated a substantial reduction in cell fitness under both prolonged aerobic and microaerobic cultivation. Unlike norvaline, isoleucine did not substitute for leucine even under harsh error-prone conditions. Our study introduces shotgun proteomics as a powerful tool in quantitative analysis of mistranslation.

Protein biosynthesis is a fundamental cellular process that occurs with an error frequency of approximately 10^−3^ to 10^−5 ^1,2. The measured error rate reflects mistakes accumulated at all steps of translation, with tRNA aminoacylation and decoding apparently being the most error-prone. Coupling of cognate amino acids and cognate tRNAs by their corresponding aminoacyl-tRNA synthetases (aaRS) defines the genetic code and provides substrates for ribosomal protein synthesis. As anticipated, these enzymes operate with a formidable accuracy to keep the error-rate of protein synthesis low. The cognate tRNA substrate is efficiently discriminated by aaRS based on the identity set of nucleotides embedded in the tRNA structure^3^. In the case of the amino acid substrates, selectivity against non-cognate amino acids is established through several mechanisms. Some aaRSs take advantage of structural or chemical differences among amino acids to discriminate against the non-cognate substrates at their ground or transition states, establishing highly specific synthetic steps in aminoacyl-tRNA (aa-tRNA) formation^4^,^5^. When a high level of structural and chemical similarity between cognate and non-cognate (named near-cognate) amino acids precludes their efficient discrimination during the synthetic steps of aminoacylation, aaRSs employ a comprehensive network of hydrolytic editing reactions to enhance the accuracy of aa-tRNA synthesis^6^,^7^.

Leucyl-tRNA synthetase (LeuRS) is responsible for decoding leucine codons in all domains of life. It binds leucine and activates it to a leucyl-AMP intermediate in the synthetic site of the Rossmann dinucleotide binding fold of the catalytic domain. The next step of aminoacylation takes place at the same active site and comprises transfer of the aminoacyl moiety to tRNA. We and others have recently shown that LeuRS from *Escherichia coli* may misrecognize the non-proteinogenic amino acid norvaline with an error frequency of 10^−2 ^8,9. Norvaline is an enzymatic side product of the leucine biosynthetic pathway^10^ and can be accumulated in *E. coli* at millimolar concentrations under microaerobic growth conditions^11^. Kinetic analysis established that norvaline mimics the cognate leucine during the synthetic steps of leucylation, providing evidence that its presence in millimolar concentrations can jeopardize the accuracy of Leu-tRNA^Leu^ synthesis. Importantly, norvaline participation in protein synthesis is prevented by the vigorous editing reaction that rapidly hydrolyses Nva-tRNA^Leu^ within the LeuRS domain dedicated for post-transfer editing, named the CP1 domain^9^,^12^.

Moreover, we showed that norvaline poses as the main threat for decoding of leucine codons^13^. The long term perception that isoleucine may substantially jeopardize the accuracy of leucylation^14^,^15^ was recently contradicted by our kinetic analyses showing that LeuRS discriminates against isoleucine extremely well during the synthetic reaction. The measured *in vitro* error frequency of isoleucine activation was only 10^−5 ^13. This led to the conclusion that the main physiological role of LeuRS proofreading is to prevent norvaline misincorporation into proteins, linking translational quality control with the bacterial adaptation mechanisms to rapidly changing oxygen environments^13^.

Translational error rates have been measured for various organisms using several approaches. Most often the analysis took advantage of a reporter protein or peptide whose level of mistranslation was independently studied through gain of protein function^16^,^17^, incorporation of a label^1^,^18^ or simply a change in a molecular mass^19^,^20^,^21^. Alternatively, some studies were performed on a larger scale in which the level of amino acid misincorporation was determined by amino acid analysis performed on the entire proteome^22^,^23^. The latter approach established the average value of cellular mistranslation, but cannot address particular issues such as: i) proteome-wide misincorporation at the specific amino acid position, ii) extent of misincorporation in different protein classes across the proteome; and iii) codon-dependent mistranslation.

To address these questions, we performed a systematic shotgun mass spectrometry (MS)-based proteomics study of amino acid misincorporation in wild-type and LeuRS editing-defective *E. coli* strains. Using high accuracy mass spectrometry and unbiased protein modification analysis^24^ we established that norvaline can be detected as a loss of a CH_2_ group (−14.01565 Da) at leucine positions. We then used a Super-SILAC (stable isotope labeling by amino acids in cell culture) approach^25^,^26^ and spectral counting to monitor misincorporation levels across several growth phases and culture conditions, to determine the percentage of norvaline (Nva) misincorporation into the *E. coli* proteome. To monitor an isobaric substitution, we performed a SILAC experiment using “heavy” leucine l-Leu-5,5,5-d3 to demonstrate that misincorporation of isoleucine at leucine positions is negligible. Finally, we showed that LeuRS editing-defective *E.coli* cells with higher levels of norvaline misincorporation show poorer fitness when co-cultured with WT-LeuRS cells. Our study presents the first systematic use of quantitative shotgun proteomics towards the global analysis of norvaline misincorporation of the *E. coli* proteome and demonstrates that high accuracy MS can be efficiently used to follow mistranslation at the proteome level.

## Results

### Near-cognate amino acids are misincorporated in the WT- and D345A-LeuRS *E. coli* strains

**Figure 1:**
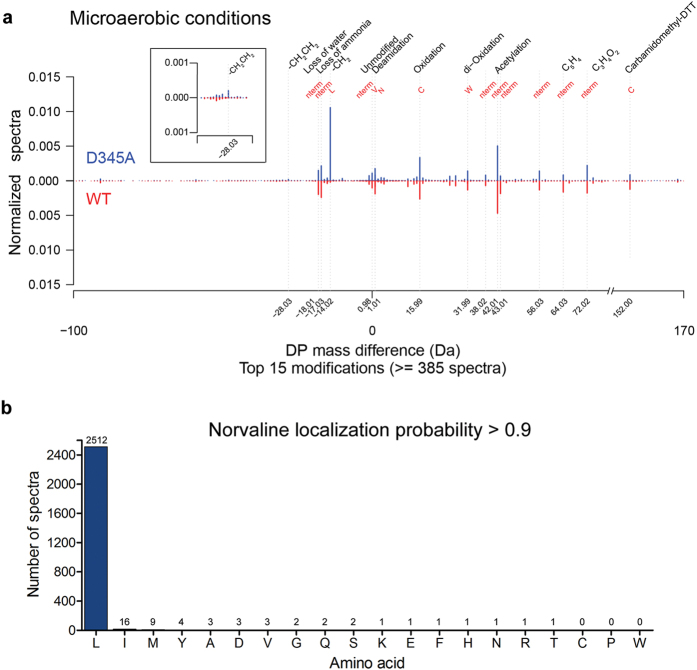
Unbiased protein modification analysis of LC-MS/MS measurements of the WT- and D345A-LeuRS MG1655 strains under microaerobic condition. (**a**) The graph depicts the frequencies of mass-differences (with respect to the unmodified peptide versions) normalized by the total number of spectra (in D345A-LeuRS 462764 and in WT 459446 spectra). The predicted modifications that correspond to the mass differences together with the modified residues are written on top of the graph. The inset shows a 5-fold enlargement of the graph area encompassing the mass difference −28.03 (corresponds to leucine substitution with α‐aminobutyrate). (**b**) Barchart illustrating the localization frequencies of norvaline sites to amino acid residues obtained with the D345A-LeuRS MG1655 strain grown in microaerobic conditions. Sites with localization probability of ≥0.9 are shown.

**Figure 2:**
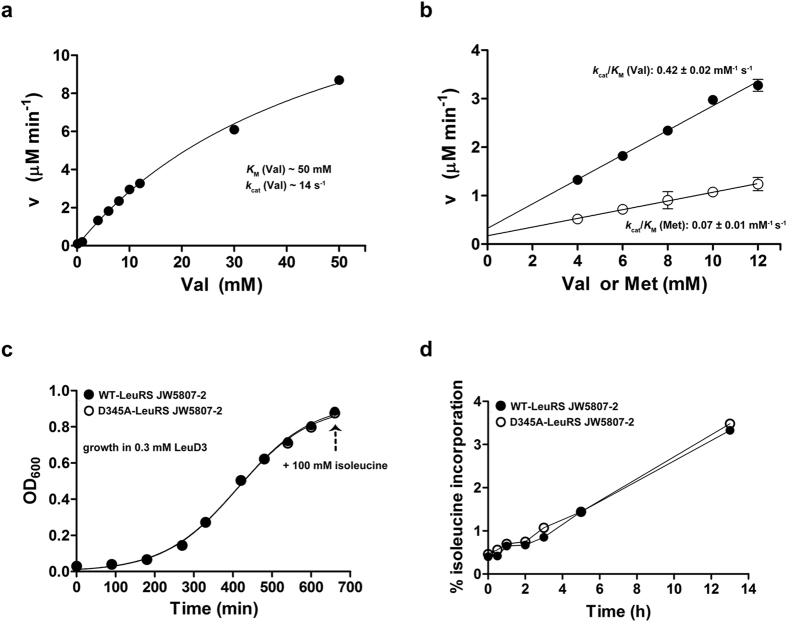
Activation of valine and methionine by WT-LeuRS. (**a**) Estimate of valine activation parameters –*k*_cat_ and *K*_M_. The separate kinetic parameters could not be accurately determined due to high *K*_M_ value and low valine solubility. (**b**) A second order rate constant *k*_cat_/K_M_ was determined to assess the catalytic efficiency of methionine and valine activation. The average value of three independent experiments for each data point is shown, and the error bar represents S.E.M. (**c**) Isoleucine misincorporation was monitored using Leu-3 SILAC. WT or D345A-LeuRS JW5807-2 strains were grown in the presence of 0.3 mM Leu-3 to enable complete labeling of the proteome. In the early stationary phase (marked with an arrow), isoleucine (100 mM final concentration) was added to both cultures and the samples were taken for LC-MS/MS analysis prior to the addition of isoleucine (denoted as 0 h), and 0.5, 1, 2, 3, 5 and 13 h after addition of isoleucine. (**d**) % of isoleucine incorporation in WT or D345A-LeuRS JW5807-2 strains.

To follow mistranslation of leucine codons under various growth conditions, we used *E. coli* MG1655 strains encoding WT- or D345A-LeuRS enzymes. The D345A-LeuRS enzyme bears a substitution of the highly conserved 345-Asp residue, which is critical for deacylation of Nva-tRNA^Leu^, in the CP1 post-transfer editing domain^9^. The D345A-LeuRS variant is generally error-prone and accumulates misaminoacylated tRNA that may participate in protein synthesis. The strains were grown under aerobic or microaerobic conditions in minimal media, their proteomes were digested into peptides and analyzed by high accuracy mass spectrometry. An overview of all performed LC-MS analyses is presented in the Supplementary Table 1. Misincorporation of near-cognate amino acids at leucine positions was addressed using an unbiased protein modification search algorithm implemented in the MaxQuant software suite (see Methods). Initially, we addressed substitution of leucine with norvaline because: i) this non-proteinogenic amino acid may significantly jeopardize the accuracy of Leu-tRNA^Leu^ synthesis *in vitro* and *in vivo*^13^, ii) norvaline represents the major target of LeuRS editing, and iii) this non-canonical amino acid substantially accumulates under microaerobic growth conditions^11^. Leucine/norvaline substitution may be described as the loss of a CH_2_ group resulting in a theoretical mass difference of −14.01565 Da. In agreement with our assumptions, the modification corresponding to an experimental mass difference of −14.01528 Da was indeed observed in samples from the D345A-LeuRS strain under microaerobic conditions (Fig. 1a), with 98% of the localized modification sites (localization probability ≥ 0.90) detected at leucine positions (Fig. 1b). The considerable level of confidence that the mass difference of -14.01528 Da describes norvaline rather than isobaric valine incorporation at the leucine sites comes from the kinetic analysis showing that LeuRS efficiently discriminates against valine at the level of ground state binding (Fig. 2a,b). The *k*_cat_ and *K*_M_ values for valine activation were estimated (Fig. 2a) to show a 1000-fold increase in *K*_M_ and a 5-fold decrease in *k*_cat_ relative to leucine activation^9^. The second order catalytic constant *k*_cat_/K_M_ for valine activation was independently determined (Fig. 2b) demonstrating again that LeuRS efficiently excludes valine with a discrimination factor of 3260 in the first step of aminoacylation reaction (i.e. the frequency of misactivation is 1 in 3260). In contrast, the discrimination factor for norvaline is only 116^9^. Furthermore, we checked for valine occurrence at leucine positions in the wild-type and D345A-LeuRS *E. coli* leucine auxotroph (JW5807-2) proteomes after prolonged cultivation during stationary phase. The leucine auxotroph strains (used for testing isoleucine misincorporation, see below) are incapable of norvaline synthesis due to disruption of the *leuB* gene by a Kan cassette. The measured misincorporation, which can be assigned only to valine, was very low (0.0072%, Supplementary Table 2). This strongly supports our conclusion that valine is well discriminated by LeuRS, providing further evidence that a mass difference of −14.01528 Da results from incorporation of norvaline under our experimental conditions. The extent of other modifications was also examined by utilizing unbiased protein modification analysis (Fig. 1, Supplementary Table 3, Supplementary Data). Substitution with methionine (theoretical mass difference 17.95642 Da) was not observed (Supplementary Table 3), which is in agreement with the kinetic data showing a very high discrimination factor of 19610 (Fig. 2b). Yet, another modification that corresponds to a mass difference −28.03112 Da was detected (Fig. 1a, inset). This modification, localized at leucine positions, was found at very low abundance almost exclusively in D345A- LeuRS strain. The experimental mass difference can be assigned to leucine substitutions with α‐aminobutyrate (theoretical mass difference: −28.03130 Da). The extent of identified leucine substitutions (about 50-fold lower than norvaline misincorporation, Supplementary Table 3) is in agreement with the *in vitro* data showing a low frequency of α‐aminobutyrate (AABA) activation by LeuRS (1 in 5500^13^). Taken together, our data demonstrate that in the absence of LeuRS editing, mistranslation of leucine sites across the proteome originates almost exclusively from norvaline misincorporation. A similar mistranslation pattern was observed for aerobic (Supplementary Fig. 1) and microaerobic growth, with more pronounced norvaline misincorporation under oxygen-deprived conditions. We further checked for post-translational modifications (PTMs) of the LeuRS that may influence substrate specificity under various growth conditions. To this end, we investigated the modification status of all LeuRS peptides identified by unbiased protein modification analysis (Supplementary Data). Detailed examination did not reveal any significant difference in detected LeuRS PTMs in all strains/conditions. Although we did not cover the whole LeuRS sequence and cannot exclude that a low-level modification escaped the detection by MS, the lack of any abundant regulatory PTMs on LeuRS indicates that *in vitro* kinetic data may be used to substantiate *in vivo* analysis.

### Leu-3 SILAC demonstrates that isoleucine is not misincorporated at leucine positions

Possible incorporation of isoleucine in the place of leucine *in vivo* cannot be assessed by unbiased protein modification analysis as Ile and Leu produce isobaric ions that have the same nominal mass but different chemical structures. To address this, we labeled the proteomes of both the WT- and D345A-LeuRS strains with “heavy” leucine, l-Leu-5,5,5-d3 (Leu-3). To enable complete proteome labeling and assess misincorporation levels, we used an *E. coli* leucine auxotroph strain (JW5807-2) and replaced the *leuS* gene which encodes for the WT-LeuRS with the variant that encodes the D345A-LeuRS enzyme incapable of hydrolysing Ile-tRNA^Leu 9^. These strains were grown in M9 media supplemented only with Leu-3. When the cells reached stationary phase, exogenous “light” (unlabeled) isoleucine was added to the medium in order to follow the misincorporation of isoleucine in place of Leu-3 (Fig. 2c). The incorporation of isoleucine was determined through a decrease in the amount of “heavy” Leu-3 label calculated as described in the Methods section. Interestingly, a small, time-dependent decrease in the Leu-3 label, that may be interpreted as isoleucine misincorporation (up to 3.5%) was seen in both the WT- and D345A-LeuRS strains (Fig. 2d). As this incorporation was present in both strains, it cannot be related to the lack of editing activity in the D345A-LeuRS strain. This decrease in Leu-3 label is most likely a consequence of contamination of the commercial isoleucine samples with (unlabeled) leucine, as previously reported^13^. Our proteome-wide analysis clearly shows that isoleucine misincorporation in place of leucine does not occur *in vivo*, as no significant isoleucine misincorporation was detected even when a non-physiologically high concentration of isoleucine was present.

### Norvaline misincorporation peaks during stationary phase in microaerobic growth conditions

**Figure 3:**
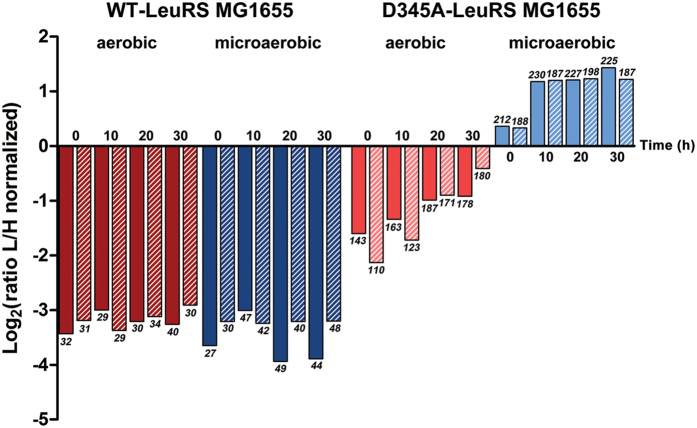
Dynamics of norvaline misincorporation during stationary phase growth of D345A- and WT-LeuRS MG1655 strains under aerobic (red bars) or microaerobic (blue bars) conditions monitored using the Super-SILAC approach. The bars represent the average values of normalized log_2_ ratio L/H for norvaline-containing peptides in each sample. Time of growth in the stationary phase is designated with numbers next to the bars, while the numbers in italic represent quantified norvaline sites. The bars with the patterns represent a biological replicate.

In order to examine the dynamics of norvaline misincorporation, we performed proteome measurements at selected time-points comprising the entry to stationary phase (denoted 0 h) and 10, 20 or 30 h cultivation in stationary phase for both the WT- and D345A-LeuRS MG1655 strain. To enable direct comparison of the strains (WT and D345A-LeuRS), growth conditions (aerobic and microaerobic) and time-points (0, 10, 20 and 30 h in stationary phase), we applied the Super-SILAC approach for relative quantification (Fig. 3, Supplementary Fig. 2). Briefly, protein extracts from 16 different samples labeled with 4,4,5,5-D_4_ l-lysine (Lys-4) were mixed to produce an internal standard, termed the Super-SILAC standard (SSS). Proteins from each 4,4,5,5-H_4_ l-Lysine (Lys-0) labeled culture were mixed separately with the Super-SILAC standard in a 1:1 ratio resulting in 16 LC-MS/MS runs per biological replicate. As expected, norvaline misincorporation was most pronounced under microaerobic conditions in the LeuRS editing-defective strain. The maximum misincorporation level was reached after 10 h of cultivation in stationary phase and was approximately 4- to 8-fold higher in microaerobic than in aerobic conditions. Misincorporation was measurable even in the WT strain, albeit at low levels (8- to 32-fold lower than in the D345A-LeuRS strain grown under aerobic and microaerobic conditions, respectively). This was independently confirmed by the unbiased protein modification analysis (Supplementary Table 3).

### The level of norvaline misincorporation reached 10% under microaerobic growth conditions

To establish the percentage of leucine residues affected by norvaline misincorporation, we used two orthogonal quantitative proteomics approaches: SILAC-based occupancy (stoichiometry) measurements at modified leucine positions, and spectral counting. To achieve this we performed a shotgun proteome measurement of SILAC “light” and “heavy” labeled cultures harvested 10 h or 30 h after entry into stationary phase (Supplementary Table 4).

We first addressed the level of mistranslation by determining occupancies at modified leucine positions using double-SILAC experiments. This approach is well established for studying PTMs^27^ and determining the extent of PTM events at localized sites. The median occupancy of norvaline sites reached up to 16% in the LeuRS strain 30 h after entry into stationary phase; however, this value was measured for a relatively low number of norvaline sites (350, or 39% of localized norvaline sites, on average), which makes it statistically uncertain. We therefore used another approach, spectral counting, to assess the extent of norvaline misincorporation (Table 1, Supplementary Table 5). The number of spectra identified for a given norvaline-containing peptide was used as a proxy for abundance. To evaluate the level of mistranslation, the number of norvaline occurrences was divided with the number of theoretical leucine positions in all detected MS/MS spectra. Using this strategy, we determined the percentage of norvaline misincorporation in place of leucine in the WT- and D345A-LeuRS strains in a time-dependent manner. The trend in norvaline misincorporation dynamics that was observed in the Super-SILAC experiments was in agreement with the misincorporation levels determined by spectral counting. The WT-LeuRS strain exhibited low misincorporation, under both aerobic and microaerobic conditions, ranging from 0.04 to 0.27% (Table 1). This error is in agreement with the experimentally established overall error rate in protein translation -1 error in 3300 peptide bonds synthesized^1^ - which further validates our analysis. The misincorporation in the D345A-LeuRS strain increased 7-fold (from 1.25% to 8.47%), under microaerobic conditions and reached 8.81% after 30 hours in stationary phase (Table 1). To increase the confidence of determined misincorporation levels, we performed a separate LC-MS/MS analysis of Lys-0 proteome samples for the WT- and D345A-LeuRS strains cultivated for 30 hours in stationary phase under microaerobic conditions. Each sample was separately analyzed using LC-MS/MS on a different mass spectrometer (Q Exactive HF) in eight technical replicates to increase the number of collected spectra. Spectral counting estimated the level of misincorporation to be 0.14 ± 0.01% in the WT- and 6.1 ± 0.1% in the D345A-LeuRS strain (Supplementary Table 6), in agreement with our analysis and reported numbers (Table 1).

### Norvaline misincorporation preferentially occurs at CTG codon sites

**Figure 4:**
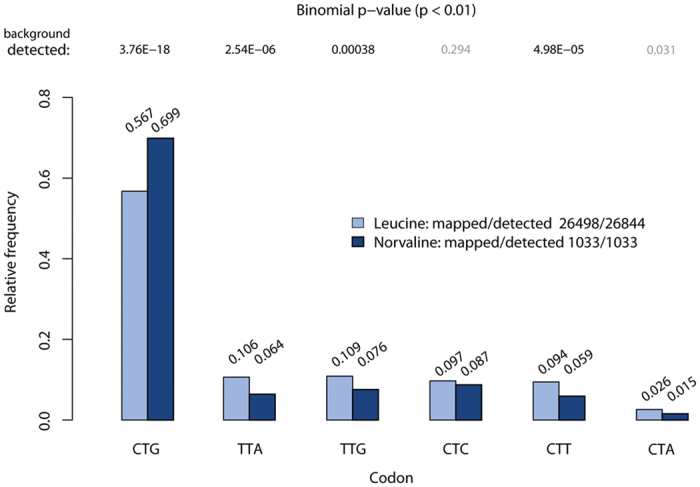
Correlation of mistranslation levels and codon usage. Frequencies of mistranslated leucine codons in the *E. coli* proteome are shown for norvaline sites with localization probability equal to 100%. The differences in the codon usage for leucine and norvaline sites were tested using a binomial test. Results from testing against the background frequencies of leucines detected in our dataset (n = 26498; label: detected) are shown.

The norvaline-containing peptides detected in our study provide a valuable dataset for exploring further features of amino acid misincorporation. We sought to explore whether norvaline misincorporation in place of leucine has a potential codon preference. To address this question, we determined the frequency of leucine codons at sites mistranslated with norvaline and compared this with the overall frequency of codons specifying leucine in the *E. coli* proteome. Only norvaline sites with localization probability of 100% were included in this dataset, thus providing a total of 1033 sites. The analysis showed that codon usage at norvaline sites follows the same trend as codon usage at leucine sites in *E. coli* (Fig. 4). When testing the statistical significance of the codon usage differences using a binomial test, we observed a statistically significant enrichment in the misincorporation of norvaline encoded at CTG codons, and statistically significant depletion of norvaline incorporation events at leucine sites encoded by TTA, TTG and CTT codons. A similar trend was observed when norvaline sites with lower localization probability were considered or when a separate test against the background frequencies of all mapped leucines in the UniProt *E. coli* database (n = 144065) was performed (Supplementary Fig. 3). Therefore, we concluded a preferential deployment of tRNA^Leu^ (CAG) isoacceptor during norvaline misincorporation. The origin of this effect is unknown but it may reflect distinct aminoacylation kinetics of the major tRNA^Leu^ isoacceptor, showing a possible interplay between amino acid and tRNA binding affinities in LeuRS. Alternatively, different interaction of Nva-tRNA^Leu^ (CAG) with the translational apparatus may influence preferential misreading of CTG codons.

### D345A-LeuRS cells show a time-dependent loss of fitness compared to the WT-LeuRS cells

**Figure 5:**
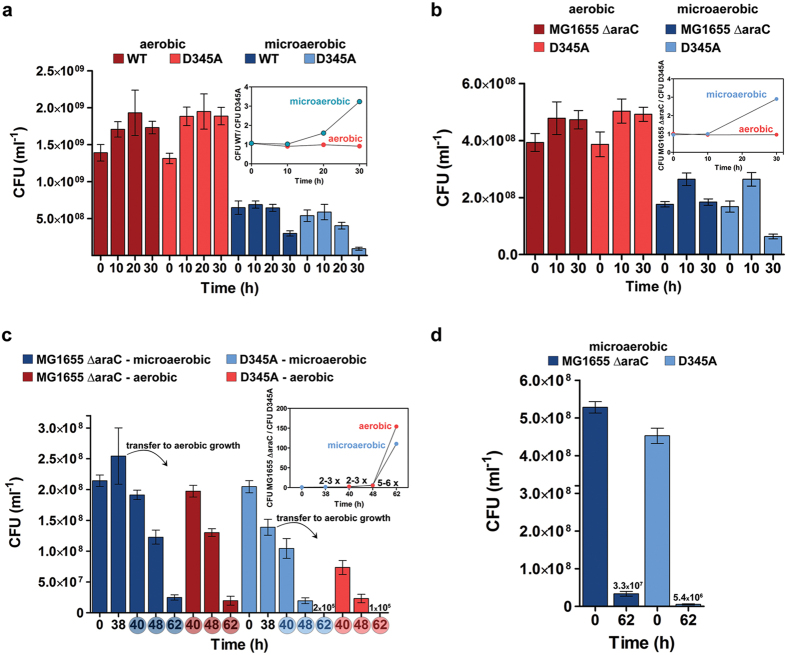
Survival of *E. coli* cells relative to the mistranslation rate. WT and editing-deficient *E. coli* (D345A-LeuRS) strains were grown under aerobic or microaerobic conditions and sampled in a time-dependent manner to assess their viability via the CFU assay. The x-axis represents the cultivation-time spent in the stationary phase (0 – early stationary phase). The red columns represent the viability of strains grown in aerobic conditions, while the blue columns represent the viability of strains grown in microaerobic conditions. The error bars represent S.E.M. The average values of the data for bars that are barely visible is presented. The insets show the time-dependent relative fitness profile calculated by dividing the viability (CFU ml^−1^) of the wild-type by the viability of the D345A strain. (**a**) WT and D345A strains were grown in separate cultures and their viability was assessed using the CFU assay. (**b**) fitness comparison of the co-cultured *ΔaraC* (containing the WT- LeuRS) and D345A-LeuRS strains. (**c**) effect of the transition from microaerobic to aerobic conditions on relative fitness of the co-cultured *ΔaraC* and D345A LeuRS- strains. Both strains were first cultured under microaerobic conditions for 38 hours after reaching the stationary phase and then transferred to aerobic conditions and cultivated for an additional 24 hrs (denoted with an arrow). The prolonged cultivation was performed under microaerobic conditions as a control, and the viability was assayed after 2, 10 and 24 hours of additional growth (40, 48, 62 hours in the stationary phase). (**d**) Δ*araC* (WT) and D345A- LeuRS strains were grown in separate cultures under microaerobic conditions and their viability assessed using the CFU assay at early stationary phase (0) and after a further 62 hours of cultivation in the stationary phase.

To explore consequences of mistranslation *in vivo*, we assessed the viability of WT- and D345A-LeuRS strains using a viable cell assay (CFU) at selected time-points (Fig. 5a). Interestingly, under aerobic conditions the viability of the editing-deficient strain (D345A-LeuRS) was indistinguishable from that of the wild-type strain (WT-LeuRS), despite its 12-fold increase in norvaline misincorporation (1.65% versus 0.14%, Table 1). However, a decrease in viability was clearly observed under microaerobic conditions, in which the D345A-LeuRS strain exhibited a 3-fold decrease in the number of viable cells after 30 h in stationary phase. At that time-point, the level of leucine substitutions with norvaline was 8.81%. This is consistent with previous work^13^, which after careful reexamination reported a fall in viability of 8-fold under prolonged microaerobic conditions. To further investigate the biological effects of mistranslation, we performed pairwise comparisons of co-cultured D345A- and WT-LeuRS strains. In these competition experiments, a *ΔaraC* derivative of the WT-LeuRS strain was used because of its inability to utilize arabinose which enables it to be distinguished from the competing strains on tetrazolium and arabinose (TA) plates. No detectable difference in fitness levels was observed between the *ΔaraC*, WT parent or the WT-LeuRS MG1655 strain under prolonged aerobic or microaerobic growth (Supplementary Fig. 4). Next, we compared the fitness of the D345A-LeuRS and *ΔaraC* strains. The competitive strains exhibited equal fitness under aerobic conditions (Fig. 5b) as the time-dependent relative fitness profile calculated by dividing the viability (expressed in CFU ml^−1^) of *ΔaraC* strain with the viability of the D345A-LeuRS strain was close to one (Fig. 5b, inset). A decreased fitness of the D345A-LeuRS strain was seen only under microaerobic conditions after 30 hours of cultivation in stationary phase, which was similar to that observed for the viability of separately grown strains (Fig. 5a). Finally, we sought to explore the effects of aerobic oxidative conditions on the relative fitness of the proteome-wide mistranslation strain in competition with the *ΔaraC* strain. The strains were equally mixed in minimal media and co-cultured under microaerobic conditions for 48 hours (38 hours of stationary phase) to seek an observable viability effect assignable to mistranslation. Aerobic conditions were then promoted by increasing the culture aeration and shaking (see Methods section). The relative fitness of the competing bacteria was estimated *via* CFU assay after 2, 10 or 24 h of growth in aerobic conditions (40, 48 and 62 hours in stationary phase). Comparison of the CFU levels revealed an increase from 6- to 150-fold in the relative fitness of the WT-LeuRS strain compared to the editing-deficient LeuRS strain after 10 or 24 hours of aerobic growth, respectively (Fig. 5c). Interestingly, the same effect was observed for the control reaction in which the cultures remained under microaerobic growth for an additional 10 or 24 hours (Fig. 5c). The relative fitness profiles (Fig. 5c, inset) implied an equal level of toxicity due to mistranslation under prolonged microaerobic or aerobic growth. To address the role of bacterial competition in the 110-fold viability drop of the D345A-LeuRS strain under extended microaerobic growth, we grew the *ΔaraC* and D345A-LeuRS strains as separate cultures. Interestingly, only a 6-fold drop in the viability of the separately grown D345A-LeuRS strain relative to the *ΔaraC* strain was observed after 62 h in stationary phase under microaerobic conditions (Fig. 5d). It appears that the larger decrease in viability of the D345A-LeuRS strain when subjected to prolonged stationary phase is predominantly a consequence of competition with the more fit *ΔaraC* strain that has the WT-LeuRS enzyme.

## Discussion

We have recently shown that the LeuRS editing domain is essential under microaerobic growth conditions in which accumulated norvaline may jeopardize the accuracy of Leu-tRNA^Leu^ synthesis. We further revealed that the commonly held view in which LeuRS frequently misactivates isoleucine *in vitro* is mistaken because it is based on measurements with impure isoleucine samples that contain traces of leucine. Our *in vivo* study revealed a similarly low sensitivity of the wild-type and LeuRS editing defective *E. coli* strains to exogenously added isoleucine. In sharp contrast, norvaline supplementation was highly toxic specifically to the strain with defective LeuRS editing^13^.

To follow up on this work we have now used shotgun quantitative mass spectrometry to address the following questions: i) does isoleucine/leucine substitution occur *in vivo* without promoting cellular toxicity, ii) what are the levels and dynamics of norvaline mistranslation under various conditions of growth and cellular viability, and iii) could some other amino acids substitute for leucine under error-prone conditions? Using the SILAC methodology to track misincorporation of isobaric amino acids isoleucine and leucine, we demonstrated that isoleucine does not replace leucine in proteins to any measurable extent, irrespective of LeuRS editing. The combined kinetic, proteomics and genetic data now provide clear evidence that isoleucine is not a substrate of LeuRS *in vitro* and *in vivo*. Our data support the notion that isoleucine editing is a redundant LeuRS activity that is dispensable for cell viability.

To study misincorporation of canonical and non-canonical amino acids into the bacterial proteome we performed a global unbiased protein modification analysis based on the correlation of unidentified MS spectra with spectra identified from unmodified peptides, as implemented in the MaxQuant software suite^24^. Similar strategies were applied before to the analysis of protein modifications^28^,^29^,^30^, but largely focusing on unbiased detection of covalent modifications and canonical amino acid substitutions. To our knowledge, this is the first application of such an approach towards the analysis of (mis)incorporation of nonproteinogenic amino acids into the proteome. Using this approach, we confirmed that mistranslation of leucine codons involves almost exclusively norvaline, which was detected by the loss of a CH_2_ group at leucine positions. We note that our MS approach could not discriminate the isobaric amino acids norvaline and valine, as the application of SILAC-labeled valine was not possible since it can be metabolized, causing the conversion of the label into other amino acids. Exact MS measurement of valine levels at leucine positions could be in principle performed by probing the sample with a high number of synthesized, stable isotope labeled and valine-containing peptide tracers^31^. However, by performing kinetic analysis and investigating valine misincorporation at leucine positions in a leucine auxotroph strain (that cannot produce norvaline), we expect that the valine misincorporation at leucine positions occurs at very low levels (Supplementary Table 2). We could effectively exclude other possible isobaric substitution, such as isoleucine /leucine by using Leu3-SILAC analysis. The discrete loss of CH_2_ groups (−14.01565 Da) enabled us also to define norvaline as a variable modification of leucine residues in MaxQuant software for subsequent measurements, which facilitated data processing.

A plateau of misincorporation of 8–9% was reached in the D345A- LeuRS strain after 10 hours in stationary phase under microaerobic conditions. However, the viability of the mistranslating strain did not fall until about 20 hours later (Fig. 5a). Thus it appears that mistranslation has a time-dependent effect on cell viability. The normal viability in early stationary phase is in line with evidence that bacteria tolerate error-prone gene expression during stationary phase, presumably to facilitate adaptation to the challenging environmental conditions^32^,^33^,^34^,^35^. However, upon prolonged incubation, accumulation of mistranslated proteins in non-proliferating cells apparently becomes a significant burden. This is consistent with findings that non-growing starved bacteria actually perform protein synthesis at a constant rate over several days in stationary phase^36^.

LeuRS editing-defective strain also exhibited time-dependent loss of the ability to compete with the wild-type strain under prolonged microaerobic cultivation during stationary phase (Fig. 5b,c). Importantly, the harmful effect of mistranslation was significantly stronger under co-culture conditions, resulting in a 110-fold drop in relative fitness of the D345A-LeuRS strain. Exposure to oxygen through better aeration in stationary phase did not promote or decrease the toxicity of mistranslation (Fig. 5c). Our data show that proteome-wide norvaline misincorporation at approximately 10% of the leucine sites significantly restricts *E. coli* long-term survival under nutrient-depleted and harsh environments. Hence, this work strongly supports a recent prediction that microbes in nutrient-poor conditions are less tolerant to mistranslation^37^. This may be a consequence of the energetic burden of enhanced proteome degradation and synthesis, substantial protein aggregation accumulation, or inability of the mistranslated proteome to completely execute the adaptive response required for keeping cells viable over longer periods of time^38^. The finding that the effect is more pronounced under strongly competitive conditions and is not promoted by transfer to aerobic conditions, suggests that mistranslation primarily influences the bacterial adaptive switch to prolonged starvation. This important life phase includes the complex strategies^39^ aimed to delay cell death. It is tempting to speculate that the aberrant protein translation encourages *E. coli* aging^40^, giving a clue why the wild-type strain outgrew the mistranslated strain that might have aged faster. Whatever the reasons of this complex behavior are, this study supports a view that mistranslation is tolerable under favorable growth conditions, but detrimental at times of prolonged starvation. As a bacterial life cycle includes long periods of starvation, this provides a clear evolutionary reason for keeping the tRNA synthetase proofreading mechanisms alive.

## Methods

### Bacterial strains

The strains used in this study were WT- and the editing-deficient D345A-LeuRS MG1655^13^ (Supplementary Table 7). The D345A-LeuRS MG1655 strain has a chromosomal *leuS* gene that encodes for a wild-type LeuRS replaced with a gene that encodes for an editing-deficient D345A-LeuRS variant. For experiments where isoleucine misincorporation was measured, *E. coli* leucine-auxotroph cells were used. Parent strain for production of the WT- and D345A-LeuRS JW5807-2 cells is the strain JW5807-2, *ΔleuB780::kan*, obtained from the Coli Genetic Stock Center (CGSC). The WT-LeuRS and D345A-LeuRS variants were produced as previously described^13^,^41^. Briefly, the replacement of the chromosomal WT *leuS* with the gene encoding for D345A-LeuRS was performed using the pKOV vector, according to the published procedures^41^. The positives were selected by their sensitivity to norvaline (10 mM norvaline is highly toxic only to editing-defective D345A-LeuRS strains). The replacement of the chromosomal gene was confirmed by sequencing. The clones insensitive to norvaline were also isolated, and the presence of the chromosomal WT *leuS* gene was confirmed by DNA sequencing. This strain was designated as WT-LeuRS JW5807-2.

### Bacterial growth and SILAC labelling

The experiments that involve aerobic bacterial growth were performed by growing 50 mL cultures (strains WT- and D345A-LeuRS MG1655, Supplementary Table 7) in minimal M9 media (1 × M9 Minimal Salts (5×, Sigma), 0.001% thiamine, 1 mM MgSO_4_, 0.1 mM CaCl_2_, 0.5% glucose) in 250 mL flasks at 37 °C, 200 rpm. The experiments that involve microaerobic growth were performed by growing 50 mL cultures in minimal M9 media in 50 mL Falcon conical tubes at 37 °C, 100 rpm. SILAC labeling for assessment of norvaline misincorporation was achieved by adding the stable isotope-labeled (“heavy”) 4,4,5,5-D_4_ l-lysine (Lys-4) in batch culture (0.0025% w/v)^42^. Separately, the cells were grown in M9 medium containing unlabeled (“light” 4,4,5,5-H_4_ l-Lysine (Lys-0), 0.0025% w/v) in otherwise the same growth conditions as the “heavy” labeled cultures. Aliquots of batch cultures (Lys-0-and Lys-4-labeled) were taken at specific time-points (early stationary phase denoted as 0 h, 10 h, 20 h and 30 h in stationary growth phase) and the cells were pelleted by centrifugation and stored at −80 °C.

The experiments involving isoleucine incorporation in place of leucine were performed in aerobic conditions by growing 60 mL cultures in 250 mL flasks (WT- and D345A-LeuRS JW5807-2 strains, Supplementary Table 7) in minimal M9 media supplemented with 0.3 mM l-Leucine-5,5,5-d3 (Leu-3) to allow for complete labelling with “heavy” leucine. After reaching stationary phase, 100 mM isoleucine (“light”) was added by dissolving isoleucine directly in the culture media. Aliquots of cultures were taken prior to isoleucine addition (designated as 0 h), and 0.5, 1, 2, 3, 5 and 13 h after isoleucine addition.

### Bacterial Lysis and Protein Extraction

The cell pellets were resuspended in the commercially available YPER lysis buffer (Thermo Scientific) supplemented with 50 μg/ml lysozyme and incubated at 37 °C for 20 min, 750 rpm. The cell suspension was briefly sonicated two times for 30 s at 40% amplitude (Sonifier I W-250, Branson) and the cellular debris pelleted by centrifugation at 13000 g for 30 min. Proteins were precipitated from the supernatant using the methanol/chloroform method. Briefly, supernatant was mixed with four volumes of methanol, one volume of chloroform and three volumes of water, and centrifuged at 4500 g for 10 min. Protein pellet formed between two organic phases was washed with methanol and resuspended in denaturation buffer containing 6 M urea, 2 M thiourea and 10 mM Tris. Protein concentrations were measured using the standard Bradford assay (Bio-Rad). The efficiency of the Lys-4 incorporation was determined by MS analysis of heavy cultures as described previously^42^. Only the extracts with 95% or higher labeling efficiency were used for downstream quantitative analysis. Protein extracts obtained from Lys-4-supplemented cultures were mixed in equal amounts to obtain the Super-SILAC standard (SSS) spiked into each growth condition. Proteins extracted from Lys-0-labeled cultures were mixed at 1:1 equimolar ratios with the SSS to produce 16 samples corresponding to 16 conditions per biological replicate. Direct pairwise comparison of proteomes was also performed using classical SILAC experiments where proteins extracted from Lys-0-labeled cultures (50 μg) were mixed at 1:1 equimolar ratios with protein extracts from Lys-4-labeled cultures (50 μg). In biological replicates of double SILAC experiments, the labels were switched in pairwise comparisons to account for any effects of the isotopically labeled lysine (Supplementary Table 4).

### In-solution protein digestion

A total of 100 μg (50 μg Lys-0 and 50 μg Lys-4) of crude protein extract in denaturation buffer was digested prior to peptide separation via isoelectric focusing, while 20 μg (10 μg SSS and 10 μg Lys-0) were digested for the Super-SILAC experiment. For isoleucine experiment and “heavy”-label incorporation checks 10 μg of protein extract was used for digestion. Proteins were reduced using 1 mM dithiothreitol (1 h incubation, 750 rpm at room temperature) followed by an alkylation step using 5.5 mM iodoacetamide (1 h incubation at room temperature in the dark). Protein pre-digestion was performed using endoproteinase Lys-C (1:100 w/w) for 3 h at room temperature. The solution was diluted with 4 volumes of 20 mM ammonium bicarbonate and supplemented with addition of endoproteinase Lys-C (1:100 w/w) for overnight digestion (approximately 16 h, 750 rpm at room temperature). Protein digestion was ceased by acidifying the solution using trifluoroacetic acid (TFA) to a final concentration of 0.1% (v/v) and desalted using C18 StageTips^43^ (see below). Samples that were further fractionated using isoelectric focusing were not acidified.

### Peptide fractionation by OFFGEL isoelectric focusing

Peptides derived from the in-solution digestion of double SILAC mixtures were separated according to their isoelectric point into 12 fractions using the 3100 OFFGEL Fractionator (Agilent Technologies) following the manufacturer’s instructions. Separation was performed on a 13-cm Immobiline DryStrips with a pH 3–10 gradient (GE Healthcare) at a maximum current of 50 μA until 20 kVh were reached. The peptide fractions were acidified with acidic solution (30% v/v acetonitrile, 5% v/v acetic acid and 10% v/v TFA) and desalted using Stage-Tips.

### Stage-tips

Prior to LC-MS/MS measurement, all peptide samples were purified by using C18 StageTips^43^. Briefly, reversed phase C18 discs (Empore) were activated with methanol and equilibrated with solvent A* (2% v/v acetonitrile and 1% v/v TFA). 10 μg of the sample was loaded onto the membrane and washed with solvent A (0.5% v/v acetic acid). The peptides were eluted with 50 μl solvent B (80% v/v acetonitrile and 0.5% v/v acetic acid) and concentrated in a vacuum centrifuge at room temperature. The volume of the peptide solution was adjusted using solvent A and 10% of solvent A*.

### LC-MS/MS Measurements

Desalted peptide samples were separated by an EASY-nLC II system (Proxeon Biosystems) coupled on-line to an Orbitrap Elite mass spectrometer (Thermo Scientific) through a nanoelectrospray ion source (Proxeon Biosystems). Chromatographic separation was performed on a 15 cm fused silica emitter with an inner diameter of 75 μm and a tip diameter of 8 μm, packed in-house with reversed-phase ReproSil-Pur C18-AQ 3 μm reversed phase resin (Dr. Maisch GmbH). The column temperature was maintained at 30 °C using an in-house integrated column. Peptides were injected onto the column with solvent A at 700 nl/min using a maximum back-pressure of 280 bar. The peptides were eluted using 79 min or 219 min (for isoleucine incorporation experiment) segmented gradient of 5–50% solvent B at a constant flow rate of 200 nl/min. The mass spectrometer was operated in a data-dependent mode, switching automatically between one full-scan and subsequent MS/MS scans of the 15 most abundant peaks (Top15 method) selected with an isolation window of 4 Th. Full scan MS spectra were acquired in a mass range from m/z 300–2000 at a target value of 1 × 10^6^ charges with the maximum injection time of 100 ms and a resolution of 120,000 (defined at m/z 400). The higher energy collisional dissociation (HCD) MS/MS spectra were recorded at a target value of 4 × 10^4^ with the maximum injection time of 150 ms at a resolution of 15,000 (defined at m/z 400) with a normalized collision energy of 35%. The masses of sequenced precursor ions were dynamically excluded from MS/MS fragmentation for 60 s. Ions with single and unassigned charge states were excluded from fragmentation selection.

Spectral counts were validated through an analysis utilizing the Q Exactive HF instrument (Thermo Scientific), which employs a different scanning scheme. Peptides were separated on the in-house packed 20 cm capillary column with 1.9 μm ReproSil-Pur C18-AQ resin (Dr. Maish GmbH) using an EASY-nLC 1000 system (Thermo Scientific) at a constant temperature of 40 °C. The pH of all the solvents was adjusted with formic acid to final 0.1%. Peptides were injected at 700 nl/min at the maximal back-pressure of 500 bar and eluted using 119 min segmented gradient of 10–50% solvent B at a flow rate of 200 nl/min. MS data was acquired using a data-dependent Top12 method. Full scan resolution was 120,000 (defined at m/z 200) and target value was 3 × 10^6^ with a maximum injection time of 25 ms. HCD fragment scans were acquired at the resolution of 30,000 (defined at m/z 200) with the target value of 1 × 10^5^, maximum injection time of 45 ms and normalized collision energy of 27%. The underfill ratio was defined at 4.5% and the intensity threshold was kept at 1 × 10^5^. Dynamic exclusion was set to 30 s and precursor ions with single, unassigned or six and higher charge states were excluded. An overview of all performed LC-MS analyses is presented in the Supplementary Table 1.

### Data processing and analysis

Acquired raw data were processed using the MaxQuant software suite (version 1.2.2.9)^24^ and the derived peak list was searched using Andromeda search engine integrated in MaxQuant^44^ against a reference *E. coli* K12 proteome (taxonomy ID 83333) obtained from UniProt (4311 protein entries; release, February 2014) and a file containing 248 common laboratory contaminants. The first search was carried out with a mass tolerance of 20 ppm, while during the main search, the mass tolerance of precursor and the fragment ions were set to 6 and 20 ppm, respectively. Multiplicity was set to two, matching the number of SILAC labels used in both “norvaline” and “isoleucine” experiments: Lys-4 or Leu-3 were specified as heavy labels, respectively. The minimum required peptide length was set to seven amino acids with the maximum of two miscleavages allowed for endoproteinase Lys-C that was set to specifically cleave at lysine C-terminus. Methionine oxidation and protein N-terminal acetylation were defined as variable modifications and carbamidomethylation of cysteines was set as a fixed modification. Additionally, in experiments where norvaline misincorporation was monitored, loss of a CH_2_ group (−14.01565 Da) from leucine was defined as a variable modification (“norvaline”) specifying leucine substitution by norvaline. During a separate unbiased search for modified peptides, “norvaline” was not specified as a variable modification, instead, dependent peptides option was enabled with mass bin size of 0.0055 Da. Peptide, protein and modification site identifications were filtered using a target-decoy^45^ approach at a false discovery rate (FDR) set to 0.01. For protein quantification, a minimum of two unmodified peptide ratio counts was required, while norvaline sites were quantified based on at least one modified peptide. To increase the number of quantified features, the “match between runs” option was enabled with a match time window set to 2 min, allowing the transfer of peptide identifications across LC-MS runs based on the retention time and accurate masses^46^.

Proteins identified by the same set of peptides were combined to a single protein group. All contaminants and reverse hits were removed during data analysis. Unbiased search for modified peptides was performed on 144 raw files derived from double SILAC experiments. Briefly, dependent peptide search implemented in MaxQuant searches all MS/MS spectra unidentified in the conventional database search (“dependent peptides”) against all already identified MS/MS spectra and reports mass differences between them. “Allpeptides.txt” MaxQuant output, table that contains a list of reported dependent peptides, was filtered for at least one MS/MS count.

For isoleucine incorporation experiment, entries from the “evidence.txt” MaxQuant output table, filtered for posterior error probability (PEP) scores of ≤0.01 with intensity reported only in the “heavy” labeling state were monitored over time. Isoleucine incorporation levels at each time-point were calculated as the difference in the numbers of all evidence entries and evidence entries having intensity only in the “heavy” state, divided by the number of all evidence entries (Supplementary Data).

To determine frequency of norvaline misincorporation, 204 raw files derived from double SILAC experiments were separately processed and analyzed with “norvaline” specified as a variable modification. A total of 48 raw files were used in the analysis of the Super-SILAC experiment. In both cases, norvaline-containing peptides were filtered for PEP scores of ≤0.01 calculated for at least one experiment. Only norvaline substitution events with a localization probability of ≥0.75 were considered as localized at the leucine positions. In the Super-SILAC experiment, normalized ratios L/H for norvaline-containing peptides were used to assess the dynamics of norvaline misincorporation (Supplementary Data). Occupancies of norvaline sites were determined as the proportion between the modified (in our case norvaline-containing) peptide and corresponding unmodified peptide using the algorithm implemented in MaxQuant based on the calculation described in Olsen *et al.*^27^ (Supplementary Data). The calculation of occupancies requires SILAC ratio of a modified peptide, the SILAC ratio of the corresponding unmodified peptide and the SILAC protein ratio. For spectral counting, MS/MS spectra were filtered for PEP scores of ≤0.01 and labeling state of either 0 for Lys-0- or 1 for Lys-4-labeled peptides. The number of occurrences of norvaline residues were counted in norvaline-containing MS/MS spectra and divided by the number of theoretical leucine positions extracted from the sequence of all MS/MS spectra.

### Kinetic analysis – activation of valine and methionine by LeuRS

To follow amino acid activation, the ATP-PP_i_ exchange assay was performed as described previously^9^,^47^,^48^. The reactions were measured at 37 °C in 50 mM Hepes (pH 7.5), 20 mM MgCl_2_, 100 μg/ml BSA, 5 mM DTT, 4 mM ATP and 1 mM [^32^P]PP_i_. WT LeuRS was present at 20 nM, and the concentrations of valine or methionine were varied to determine steady-state parameters. Separately, when saturation with the amino acid could not be reached, a second order catalytic rate constant *k*_cat_/*K*_M_ was determined from the slope of the linear portion of the velocity *versus* amino acid concentration progress curve.

### Codon usage analysis

For analysis of codon usage all *E. coli* proteins (UniProt database) were mapped to the *E. coli* reference genome (NC_000913) using tblastn^49^ in order to retrieve the genomic coordinates of each leucine in the *E.coli* proteome. Following this approach 99.23% of all leucines mapped to the six codons known to encode for this amino acid (Supplementary Fig. 5). The remaining 0.77% of leucines could not be mapped correctly to the genome either due to an incomplete mapping of the corresponding protein sequence in the tblastn analysis or due to annotation discrepancies between UniProt and Refseq. Codons of each leucine/norvaline site detected in our dataset were extracted and included in the analysis if the localization probability for each norvaline site was 100% (n = 1033). Frequencies of the six codons were calculated and the two-sided Binomial test (R-function ‘binom.test’) was applied to determine differences in the codon usage between leucine and norvaline. A separate test against the background frequencies of all mapped leucines in the UniProt *E. coli* database (n = 144065) and all detected leucines in our dataset (n = 26498) was performed (Supplementary Fig. 3).

### Assessment of cell viability

The WT-LeuRS MG1655 and the editing deficient D345A-LeuRS MG1655 strain^9^ were inoculated in M9 minimal media supplemented with 0.5% glucose from a single colony and grown to saturation at 37 °C. The cultures were diluted a starting OD_600_ of 0.025. Aerobic conditions were ensured by shaking 50 mL media in 250 mL flasks at 200 rpm. Microaerobic conditions were promoted from the beginning of the microaerobic growth experiment by cultivating the bacteria in 50 mL Falcon tubes filled with 50 mL of minimal media and shaking at 100 rpm. The colony forming unit (CFU) assay was performed at certain time-points to assess viability of the bacteria. The cultures were diluted in phosphate buffered saline at appropriate dilutions to ensure the number of colonies in a range from 30 to 800. The plates were incubated for approximately 16 h at 37 °C before counting. The competition experiments were performed using an *E. coli* strain that cannot utilize arabinose^50^ as a carbon source - MG1655 ΔaraC. This strain was derived from JW0063-1 strain (obtained from CGSC) by transfer of the arabinose utilization marker Δ(araD-araB)567 ΔaraC771::kan using P1 phage transduction into the MG1655 strain. The kanamycin cassette was further removed by standard procedures to produce a Kan sensitive strain and achieve the most similar genetic background to D345A-MG1655. Competition assays were performed to estimate the relative fitness of two *E. coli* strains. The strains were grown separately in M9 media until they reached saturation and the competition experiment was started by diluting each strain to a starting 0.015 OD_600_ in the same media (total OD_600_ 0.030). The viability of the competitor strains was estimated using the CFU assay on indicator TA plates (tetrazolium and arabinose plates) which enabled distinction of the strains (MG1655 ΔaraC as the WT LeuRS strain and D345A-LeuRS MG1655 strain) through an arabinose utilization marker. Plated culture dilutions (100 μL were plated) that allow for a colony number between 30 and 800 were taken as representative for colony counting and calculations of CFU per milliliter of culture.

## Additional Information

**Accession codes:** The mass spectrometry proteomics data have been deposited to the ProteomeXchange Consortium (http://proteomecentral.proteomexchange.org/) via the PRIDE partner repository^51^ with the dataset identifier PXD003468.

**How to cite this article**: Cvetesic, N. *et al.* Proteome-wide measurement of non-canonical bacterial mistranslation by quantitative mass spectrometry of protein modifications. *Sci. Rep.* **6**, 28631; doi: 10.1038/srep28631 (2016).

## Supplementary Material

Supplementary Information

Supplementary Data

## Figures and Tables

**Table 1:** Levels of leucine to norvaline substitution determined using spectral counting^a^.

Sample^b^	Nr. Nva	Nr. Leu^c^	Nva/Leu (%)^d^
WT A 0 h	41	37481	0.11
WT A 10 h	54	37570	0.14
WT MA 0 h	22	56910	0.04
WT MA 10 h	71	44175	0.16
WT MA 30 h	124	45457	0.27
D345A A 0 h	345	39552	0.87
D345A A 10 h	795	48266	1.65
D345A MA 0 h	668	53411	1.25
D345A MA 10 h	4332	51124	8.47
D345A MA 30 h	4979	56504	8.81

^a^Norvaline and leucine occurrences were counted in all identified MS/MS spectra (filtered for PEP score ≤ 0.01) and summed for each SILAC labeling state and experiment (Supplementary Data).

^b^The number of norvalines and leucines are a sum of two biological replicates.

^c^Theoretical number of leucines extracted from the sequence of all identified peptides.

^d^The percentage of leucine substitutions with norvaline is calculated by dividing the number of detected norvalines with the number of theoretical leucines.
