# Supplementary material for: Proteome-wide measurement of non-canonical bacterial mistranslation by quantitative mass spectrometry of protein modifications
Source: Sci Rep. 2016 Jul 5;6:28631. doi: 10.1038/srep28631 (PMC4932531; doi:10.1038/srep28631)
Supplement: Supplementary Information [file srep28631-s1.doc]

**Supplementary Information for**

Proteome-wide measurement of non-canonical bacterial mistranslation

by quantitative mass spectrometry of protein modifications

Nevena Cvetesic^1*^, Maja Semanjski^2*^, Boumediene Soufi^2^, Karsten Krug^2^,

Ita Gruic-Sovulj^1‡^, Boris Macek^2‡^

1) Chemistry Department, Faculty of Science, University of Zagreb, Croatia

2) Proteome Center Tuebingen, University of Tuebingen, Germany

*These authors have equally contributed to this work

^‡^ Correspondence to:

Prof. Ita Gruic Sovulj, Ph.D.

Chemistry Department

Faculty of Science

University of Zagreb

Croatia

tel: +385-1-4606232

e-mail: [gruic@chem.pmf.hr](mailto:gruic@chem.pmf.hr)

Prof. Dr. Boris Macek

Interfaculty Institute for Cell Biology

University of Tuebingen

Auf der Morgenstelle 15

72076 Tuebingen

Germany

e-mail: [boris.macek@uni-tuebingen.de](mailto:boris.macek@uni-tuebingen.de)

**Supplementary Tables**

**Supplementary Table 1.** Overview of all performed LC-MS analyses

| Experiment | Labeling | Fractionation | LC-MS/MS runs (gradient) | Total PSM | Peptides | Instrument | Quantification |
| --- | --- | --- | --- | --- | --- | --- | --- |
| Dependent peptide analysis | Lys-0/Lys-4 | OFFGEL | 144^a^ (79 min) | 401018^b^ | 12834 | Orbitrap Elite | spectral counting |
| Isoleucine incorporation | Leu-3/Ile-0^c^ | N/A | 14 (219 min) | 235811 | 13281 | Orbitrap Elite | SILAC |
| Super-SILAC | Lys-0/Lys-4 | N/A | 48 (79 min) | 430409 | 9909 | Orbitrap Elite | SILAC |
| Mistranslation level | Lys-0/Lys-4 | OFFGEL | 204 (79 min) | 535241 | 12849 | Orbitrap Elite | spectral counting |
| Validation of mistranslation level | Lys-0 | N/A | 16 (119 min) | 256950 | 11016 | QExactive HF | spectral counting |

^a^These LC-MS/MS runs were also included in the analysis of mistranslation level (204 runs).
^b^1351872 spectra in allpeptides.txt were used for unbiased protein modification analysis.

^c^Ile-0 represents unlabeled isoleucine.

**Supplementary Table 2.** Level of leucine to valine substitutions determined using spectral counting in the experiment where isoleucine mistranslation was followed in Leu-3 labeled *E. coli* leucine auxotroph strain^a^.

| Total spectra | Val spectra^b^ | Leu spectra | Nr. Val | Nr. Leu^c^ | Nva/Leu (%)^d^ |
| --- | --- | --- | --- | --- | --- |
| 235924 | 27 | 192756 | 29 | 403230 | 0.00719 |

^a^Valine and leucine occurrences were counted in all identified MS/MS spectra (filtered for PEP score ≤ 0.01) and summed.

^b^Leucine to valine substitution was defined as a variable modification with the mass difference of -17.03448 Da relative to labeled Leu-3.

^c^Theoretical number of leucines extracted from the sequence of all identified peptides.

^d^The percentage of leucine substitutions with valine is calculated by dividing the number of detected valines with the number of theoretical leucines.

**Supplementary Table 3.** Unbiased protein modification analysis^a^.

| condition | Δm observed /Da | Δm theoretical/Da | Δm/ppm^b^ | Nr. spectra D345A | Nr. spectra WT | position^c^ | modification^d^ |
| --- | --- | --- | --- | --- | --- | --- | --- |
| MA | -14.01528 | -14.01565 | 26.40 | 4896 | 102 | Leu | Leu→Nva |
| MA | -28.03112 | -28.03130 | 6.42 | 97 | 5 | Leu | Leu→AABA |
| MA | - | 17.95642 | - | - | - | - | Leu→Met |
| A | -14.01528 | -14.01565 | 26.40 | 548 | 23 | Leu | Leu→Nva |

^a^The spectra containing a specific modification were counted separately for the WT or the D345A-LeuRS strain and for microaerobic (MA) or aerobic (A) growth.
^b^Δm/ppm represents the difference between the observed and theoretical Δm.

^c^Amino acid containing modification calculated based on the spectra with the positional probability of ≥0.90 in D345A-LeuRS strain.

^d^Modification that corresponds to the observed and theoretical mass difference.

**Supplementary Table 4. Samples for double SILAC experiments.**

| SILAC^a^ |  | | Sample 1 | | | Sample 2 | |
| --- | --- | --- | --- | --- | --- | --- | --- |
|  | Strain^b^ | Cond. | | Time/h | Label | Time/h | Label |
| 1 | WT | A | | 10 | Lys-4 | 0 | Lys-0 |
| 2 | WT | MA | | 10 | Lys-4 | 0 | Lys-0 |
| 3 | WT | MA | | 30 | Lys-4 | 0 | Lys-0 |
| 4 | D345A | A | | 10 | Lys-4 | 0 | Lys-0 |
| 5 | D345A | MA | | 10 | Lys-4 | 0 | Lys-0 |
| 6 | D345A | MA | | 30 | Lys-4 | 0 | Lys-0 |
| 7 | WT rep. | A | | 10 | Lys-0 | 0 | Lys-4 |
| 8 | WT rep. | MA | | 10 | Lys-0 | 0 | Lys-4 |
| 9 | WT rep. | MA | | 30 | Lys-0 | 0 | Lys-4 |
| 10 | D345A rep. | A | | 10 | Lys-0 | 0 | Lys-4 |
| 11 | D345A rep. | MA | | 10 | Lys-0 | 0 | Lys-4 |
| 12 | D345A rep. | MA | | 30 | Lys-0 | 0 | Lys-4 |

^a^Double SILAC experiments were performed by direct mixing of proteome samples (WT or D345A LeuRS strain) grown under aerobic (A) or microaerobic (MA) conditions (cond.). Specific time-points, labeled with Lys-4 or Lys-0, were mixed with the 0 h time point labeled with Lys-0 or Lys-4, respectively, of the corresponding strain and condition.

^b^WT represents WT-LeuRS MG1655 and D345A represents D345A-LeuRS MG1655 strain. Rep. is short for replicate (biological replicate).

**Supplementary Table 5.** Level of leucine to norvaline substitutions determined using spectral counting^a^.

| **Sample**^b^ | **Total spectra** | **Nva spectra** | **Leu spectra** | **Nr. Nva** | **Nr. Leu^c^** | **Nva/Leu (%)**^d^ |
| --- | --- | --- | --- | --- | --- | --- |
| WT A 0 h | 16232 | 25 | 12907 | 25 | 24253 | 0.10 |
| WT A 0 h rep. | 9304 | 16 | 7270 | 16 | 13228 | 0.12 |
| WT A 10 h | 7567 | 14 | 5919 | 14 | 11156 | 0.13 |
| WT A 10 h rep. | 17468 | 36 | 13915 | 40 | 26414 | 0.15 |
| WT MA 0 h | 27361 | 11 | 22053 | 11 | 42702 | 0.03 |
| WT MA 0 h rep. | 9793 | 10 | 7722 | 11 | 14208 | 0.08 |
| WT MA 10 h | 10893 | 20 | 8729 | 20 | 16306 | 0.12 |
| WT MA 10 h rep. | 18602 | 51 | 14948 | 51 | 27869 | 0.18 |
| WT MA 30 h | 8846 | 37 | 7043 | 37 | 13015 | 0.28 |
| WT MA 30 h rep. | 21150 | 85 | 17009 | 87 | 32442 | 0.27 |
| D345A A 0 h | 19709 | 278 | 15710 | 278 | 29464 | 0.94 |
| D345A A 0 h rep. | 7232 | 67 | 5578 | 67 | 10088 | 0.66 |
| D345A A 10 h | 11551 | 386 | 9207 | 386 | 17620 | 2.19 |
| D345A A 10 h rep. | 19908 | 409 | 15982 | 409 | 30646 | 1.33 |
| D345A MA 0 h | 22181 | 302 | 17909 | 302 | 34839 | 0.87 |
| D345A MA 0 h rep. | 11727 | 361 | 9513 | 366 | 18572 | 1.97 |
| D345A MA 10 h | 15460 | 2293 | 12793 | 2395 | 24765 | 9.67 |
| D345A MA 10 h rep. | 18941 | 1870 | 15559 | 1937 | 30651 | 6.32 |
| D345A MA 30 h | 13147 | 2235 | 10698 | 2337 | 20473 | 11.42 |
| D345A MA 30 h rep. | 22838 | 2503 | 18631 | 2642 | 36031 | 7.33 |

^a^ Norvaline and leucine occurrences were counted in all identified MS/MS spectra (filtered for PEP score ≤ 0.01) and summed for each SILAC labeling state and experiment. In the first biological replicate, spectra of Lys-0- and Lys-4-labeled peptides were assigned to 0 h and 10 or 30 h time point, respectively. In the second biological replicate, the labels were reversed and Lys-0- and Lys-4-labeled peptides were assigned to 10 or 30 h time point, or 0 h time point, respectively.

^b^Two biological replicates are presented (rep. - replicate).

^c^Theoretical number of leucines extracted from the sequence of all identified peptides.

^d^The percentage of leucine substitutions with norvaline is calculated by dividing the number of detected norvalines with the number of theoretical leucines.

**Supplementary Table 6.** Validation of mistranslation level measured on Q Exactive HF instrument^a^.

| **Sample** | **Technical replicate** | | **Nr. Nva** | **Nr. Leu** | **Nva/Leu (%)**^b^ |
| --- | --- | --- | --- | --- | --- |
| WT MA 30 h Lys-0 | 1 | | 32 | 23639 | 0.14 |
|  | 2 | | 34 | 24861 | 0.14 |
|  | 3 | | 38 | 25327 | 0.15 |
|  | 4 | | 30 | 25524 | 0.12 |
|  | 5 | | 38 | 25413 | 0.15 |
|  | 6 | | 33 | 24704 | 0.13 |
|  | 7 | | 31 | 25001 | 0.12 |
|  | 8 | | 36 | 25046 | 0.14 |
| D345A MA 30 h Lys-0 | 1 | | 1620 | 25654 | 6.31 |
|  | 2 | | 1606 | 26547 | 6.05 |
|  | 3 | | 1631 | 26770 | 6.09 |
|  | 4 | | 1589 | 26389 | 6.02 |
|  | 5 | | 1548 | 25452 | 6.08 |
|  | 6 | | 1596 | 26275 | 6.07 |
|  | 7 | | 1592 | 26163 | 6.08 |
|  | | 8 | 1555 | 26067 | 5.97 |

^a^Eight technical replicates of only Lys0-labeled cultures were measured on Q Exactive HF instrument.

^b^Mistranslation level was calculated using spectral counting as described in Supplementary Table 3.

**Supplementary Table 7.** Bacterial strains used in this study.

| **Strain** | **Relevant phenotype** | **Experimental use** | **Source and reference** |
| --- | --- | --- | --- |
| MG1655 |  | parent strain and control strain | CGSC |
| D345A-LeuRS MG1655 | D345A LeuRS | norvaline misincorporation | Cvetesic *et al* 2014 (1) |
| WT-LeuRS MG1655 | WT LeuRS | norvaline misincorporation | Cvetesic *et al* 2014 (1) |
| JW5807-2 | Leu auxotroph | parent strain | CGSC |
| D345A-LeuRS JW5807-2 | D345A LeuRS and Leu auxotroph | isoleucine misincorporation | this study |
| WT-LeuRS JW5807-2 | WT LeuRS and Leu auxotroph | isoleucine misincorporation | this study |
| JW0063-1 | cannot metabolise arabinose | parent strain | CGSC |
| MG1655 ΔaraC | cannot metabolise arabinose | competition assays | this study |

**Supplementary Figures**

**Supplementary Figure 1.**

**
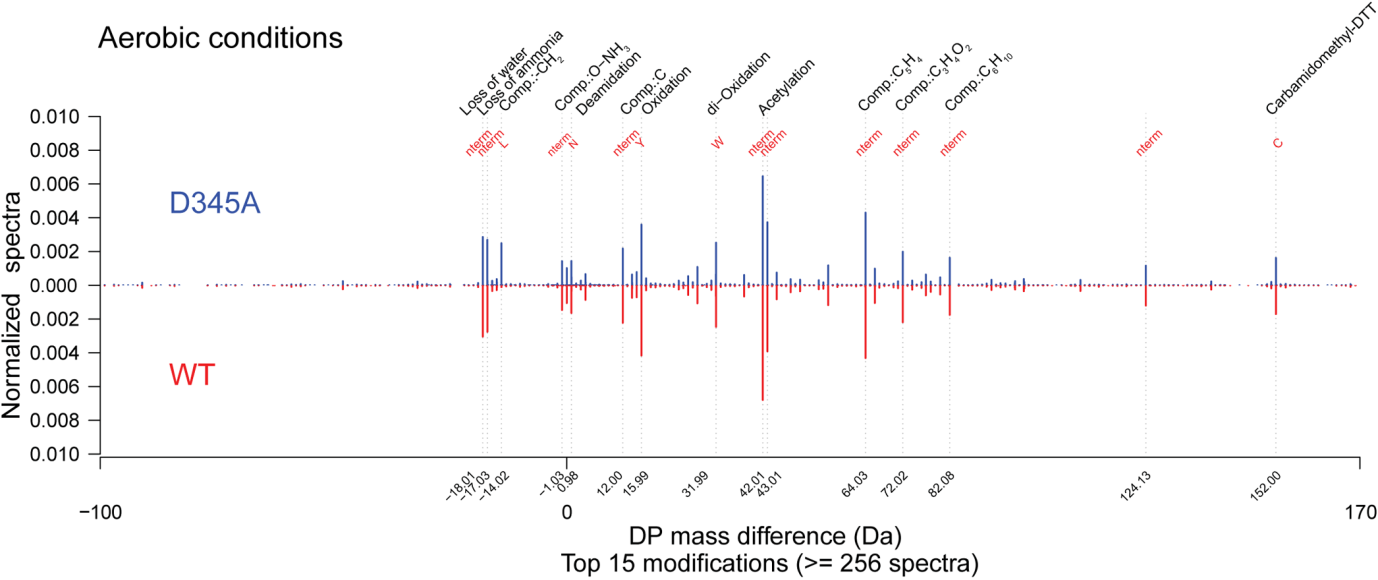
**

Dependent peptide analysis of LC-MS/MS measurements of WT- and D345A-LeuRS MG1655 strains grown in aerobic conditions. The mass-difference frequencies are normalized with the total number of spectra (in D345A-LeuRS 220522 and in WT 209140 spectra). The predicted modifications that correspond to the mass differences are written on top of the graph (Comp. stands for composition), and below are written the localizations in red.

**Supplementary Figure 2.**

**
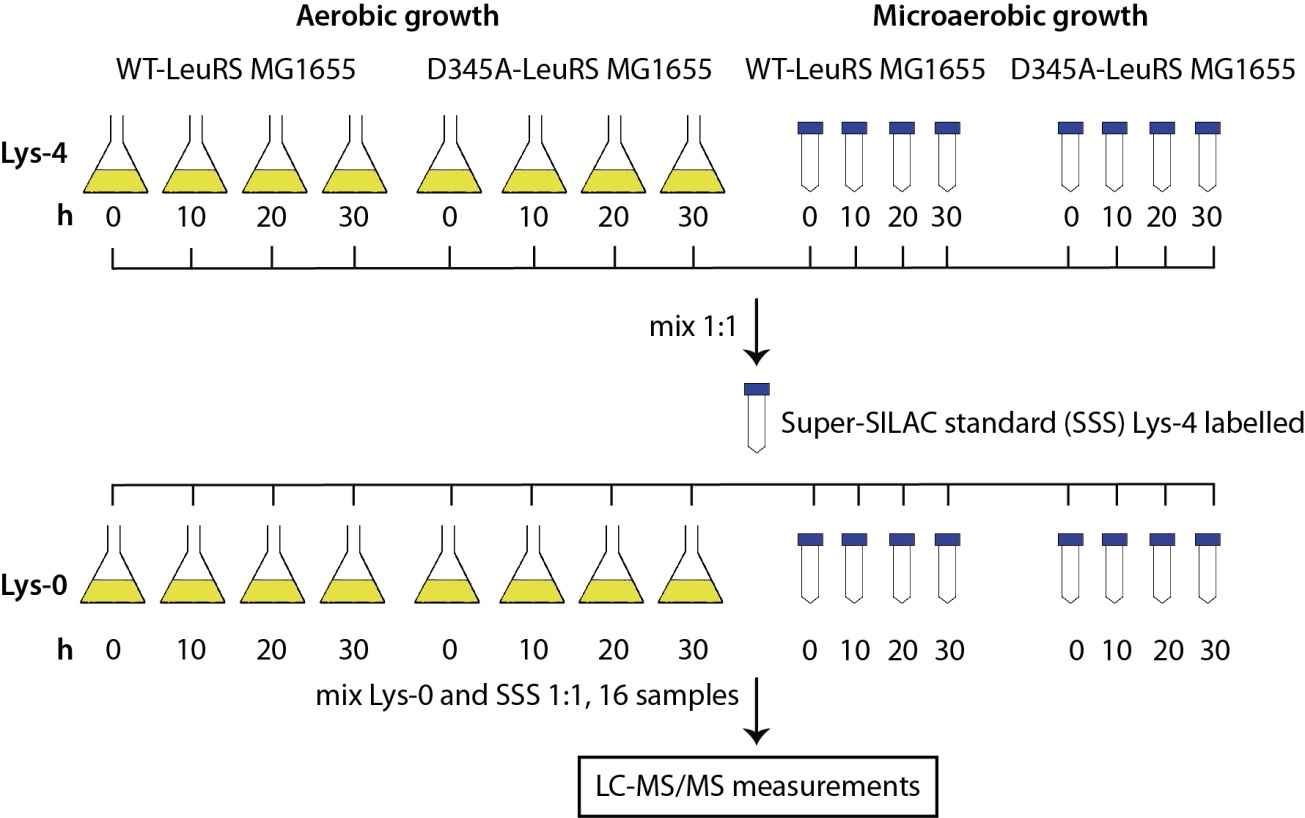
**

Design of the Super-SILAC experiment for monitoring norvaline incorporation dynamics. WT-LeuRS or D345A-LeuRS MG1655 strains were grown in aerobic and microaerobic conditions in M9 media supplemented with Lys-4. The cultures were sampled in the early stationary phase (0 h) and 10, 20 or 30 h in the stationary phase. All extracted proteins (16 samples) were mixed in 1:1 ratio to form a Super-SILAC standard. The cultures were grown in the same conditions albeit in M9 media supplemented with Lys-0. Each Lys-0 protein sample was mixed with the Super-SILAC standard in 1:1 ratio, yielding in total 16 samples per biological replicate.

**Supplementary Figure 3.**


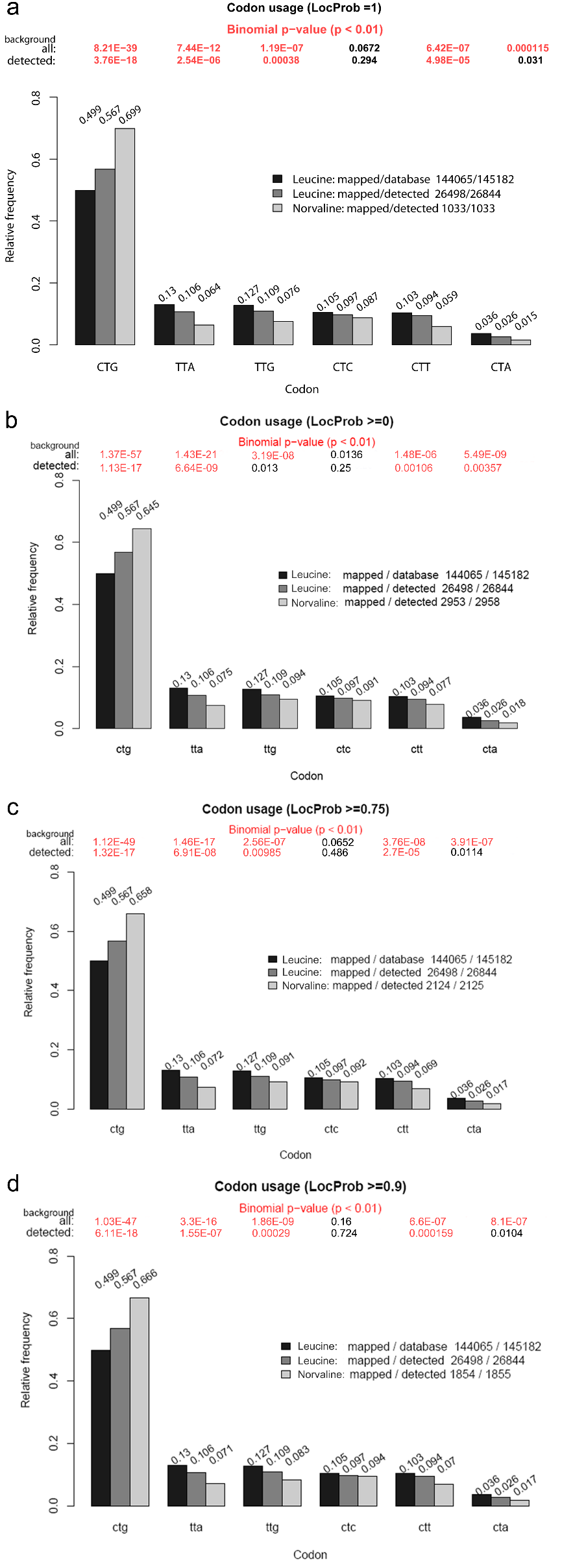


Correlation of mistranslation levels and codon usage. Frequencies of mistranslated leucine codons in *E. coli* proteome are shown for norvaline sites with a) localization probability = 1, b) localization probability >= 0, c) localization probability >= 0.75, d) localization probability >= 0.9. The differences in the codon usage for leucine and norvaline sites were tested using a binomial test. Separate results from testing against the background frequencies of all mapped leucines in the UniProt *E. coli* database (n=144065; label: all) and all detected leucines in our dataset (n=26498; label: detected) are shown.

**Supplementary Figure 4.**


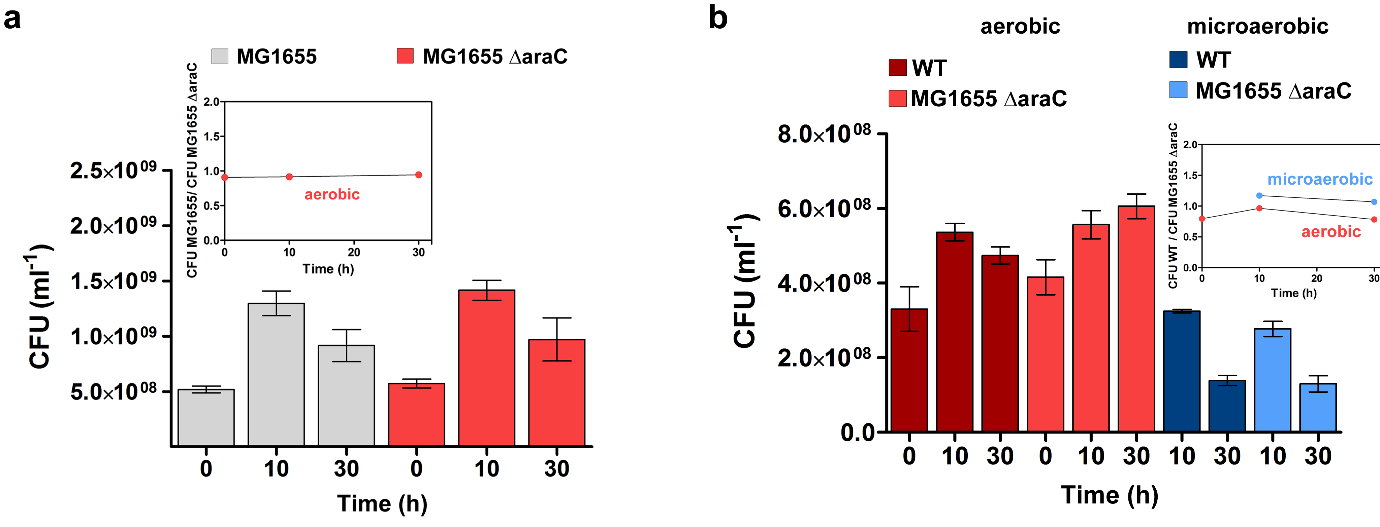


Bacterial competition assay. (a) fitness comparison of the co-cultured MG1655 ΔaraC and the parent MG1655 strain. (b) fitness comparison of the co-cultured WT-LeuRS MG1655 strain and MG1655 *ΔaraC* strains. These tests confirm the neutrality of the arabinose utilization marker. The error bars represent S.E.M.

**Supplementary Figure 5.**

**
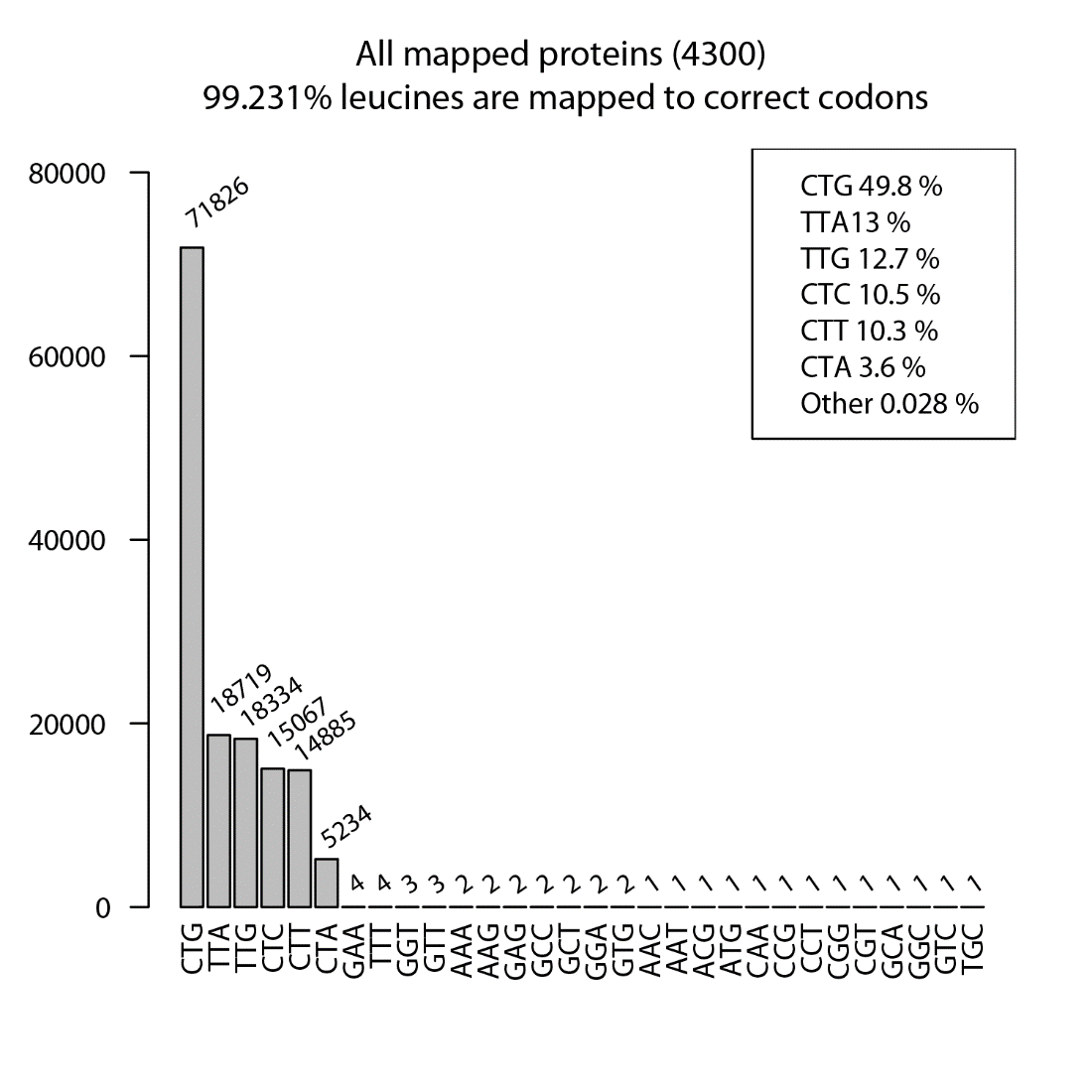
**

Mapping of leucines to six leucine codons (CTG, TTA, TTG, CTC, CTT, CTA).

**Supplementary references**

1. Cvetesic, N., Palencia, A., Halasz, I., Cusack, S., and Gruic-Sovulj, I. (2014) The physiological target for LeuRS translational quality control is norvaline. *EMBO J* 33, 1639-1653
